# PD-1/PD-L1 inhibitors for advanced or metastatic cervical cancer: From bench to bed

**DOI:** 10.3389/fonc.2022.849352

**Published:** 2022-10-14

**Authors:** Weijia Huang, Jiewei Liu, Kai Xu, Huilin Chen, Ce Bian

**Affiliations:** ^1^ Lung Cancer Center, West China Hospital, Sichuan University, Chengdu, China; ^2^ West China School of Medicine, Sichuan University, Chengdu, China; ^3^ Department of Gynecology and Obstetrics, Key Laboratory of Obstetrics and Gynecologic and Pediatric Diseases and Birth Defects of Ministry of Education, West China Second University Hospital, Sichuan University, Chengdu, China

**Keywords:** cervical cancer, tumor microenvironment, PD-1, PD-L1, immune checkpoint inhibitor

## Abstract

Advanced or metastatic cervical cancer has a poor prognosis, and the 5-year overall survival is <5% with conventional radiotherapy and chemotherapy. Immunotherapy, particularly immune checkpoint inhibitors (ICIs), achieved initial success in advanced solid tumors, while their efficacy and safety in advanced or metastatic cervical cancer remains to be explored. Previous studies found high-risk HPV infection and elevated PD-L1 expression in cervical precancerous lesions and squamous cell carcinoma. Meanwhile, elevated PD-L1 expression, high cytotoxic T lymphocyte infiltration, and abnormal cytotoxic T lymphocyte function might benefit inflammation infiltration for ICIs in the tumor microenvironment. Patients with HPV infection, squamous cell carcinoma, advanced stage, large tumor size, poor differentiation, metastatic disease, history of multiple childbirth and abortion, or a previous history of receiving chemotherapy might be associated with positive PD-L1 expression. Although there is no correlation between PD-L1 expression and prognosis using conventional radiotherapy, patients with high PD-L1 expression have a poorer prognosis. Several clinical studies demonstrate preliminary safety and efficacy for PD-1/PD-L1 inhibitors, and the exploration of combination strategies such as immunotherapy combined with chemotherapy, radiotherapy, anti-angiogenesis therapy, or dual ICIs is ongoing. This paper systematically reviews PD-L1 expression patterns and their relationship with prognosis, along with reported and ongoing clinical trials of PD-1/PD-L1 inhibitors in cervical cancer to clarify the prospect of ICIs for cervical cancer from bench to bed.

## Introduction

Cervical cancer is the fourth most common malignant tumor and a leading cause of cancer death in females, especially in regions of comparatively low human development index, which is a comprehensive index on the life expectancy and the level of education and incomes (1, 2). With the popularization of human papillomavirus (HPV) vaccines and the implementation of cancer screening potentially reducing the incidence and mortality of cervical cancer, nearly 15% of patients are staged with metastatic disease at diagnosis (3, 4). Patients with early-stage cervical cancer could be treated with radical surgical resection or radiotherapy with a favourable 5-year overall survival (OS), while the treatment modality is limited to chemoradiotherapy with or without anti-angiogenesis agents for advanced or metastatic cervical cancer, the survival rate is relatively low (5–8). The 5-year OS of early-stage cervical cancer with surgical resection reaches 90% (9), while it declines to <5% among patients with advanced/metastatic cervical cancer (6). Thus, novel and effective treatments are urgently needed.

Immunotherapy is a recently-emerged anti-tumor treatment that eliminates the immunosuppression of the immune microenvironment and mobilizes the immune system to confront tumor cells. Accordingly, the immune system maintains a dynamic balance between activation and suppression so that B7, expressed on T cells, can bind to MHC I molecules on antigen-presenting cells to activate T cells to induce an immune reaction (10). Meanwhile, when the programmed death receptor-1 ligand (PD-L1), an inhibition pathway molecule expressed on the surface of antigen-presenting cells or tumor cells, binds to programmed death receptor-1 (PD-1), expressed on T cells, then T-cell activity might be suppressed along with the immune system (11). The co-stimulation and suppression pathways antagonize each other to maintain the immune system in a dynamic equilibrium. The elevated expression of PD-L1 on tumor cells leads to a suppressed immune microenvironment, resulting in suppressed T-cell function and failure of tumor cell elimination. In this way, PD-1/PD-L1 inhibitors suspend immunosuppression and reactivate the immune system by blocking the PD-1/PD-L1 pathway to eliminate tumor cells (12).

Immunotherapy has made promising progress in treating several solid tumors, including non-small cell lung cancer (NSCLC), melanoma, and urothelial carcinoma (13). Meanwhile, increasing interest has been drawn to advanced or metastatic cervical cancer in hopes that immunotherapy could promote survival. The phase I Keynote-001 trial showed staggering efficacy in NSCLC with an estimated 5-year OS of treatment-naïve patients of 23.2%, compared to a historical level of 5.5% in those with distant metastatic diseases (14). It brought hope to patients with metastatic malignancies that they might also greatly benefit from this novel immunotherapy. However, the administration of immunotherapy in cervical cancer was still at an exploratory stage. Since Pembrolizumab was first clinically approved by the FDA in September 2014 (15), only six clinical trials concerning PD-1/PD-L1 inhibitors have published preliminary results for cervical cancer until 2020 (16–21), most being single-arm clinical trials on a small scale.

Nevertheless, immune checkpoint inhibitors (ICIs) tend to show great potential in advanced or metastatic cervical cancer. In 2017, the KEYNOTE-028 trial first showed the favourable safety and efficacy outcomes of single agent of Pembrolizumab in PD-L1–positive advanced cervical cancer that the median overall survival (mOS) was 11 months with acceptable incidence of severe adverse events (AEs) as 20.8% (16). The KEYNOTE-158 trial also confirmed durable anti-tumor activity and showed that those with PD-L1-positive cervical cancer might benefit from Pembrolizumab, in which the mOS was 11 months in the PD-L1-positive population (versus 9.4 months in the total population), and all patients that responded to Pembrolizumab were PD-L1-positive (17). Afterwards, dozens of trials are in progress, ranging from multi-line treatment to first-line treatment, from phase II single-arm studies to phase III randomized controlled studies (6, 11, 22). Several clinical trials are currently exploring a combined treatment strategy involving immunotherapy, including vaccines, adoptive cell therapy, and immunological checkpoint inhibitors (23, 24). Here we overview the association between cervical cancer and PD-1/PD-L1 expression, along with clinical evidence to illustrate the perspectives of PD-1/PD-L1 ICIs in advanced or metastatic cervical cancer.

## Immunological tumor microenvironment of cervical cancer and its relationship with immunotherapy

The tumor immuno-microenvironment plays an essential part in cancer progression (25), and the immune system also plays a vital role in eliminating and controlling early-stage tumor development, including innate immunity and adaptive immunity (26). T cell activation requires the interaction of various cytokines, and T cells can express a variety of co-stimulation receptors, such as CD28, OX40, etc. (27). Receptors, binding to related cytokines, can be activated to promote the proliferation and differentiation of T cells. Meanwhile, suppressive pathways that negatively regulate T cell activity play an essential role in balancing immune activity *in vivo*, among which the PD-L1/PD-1 pathway is important.

**Figure 1:**
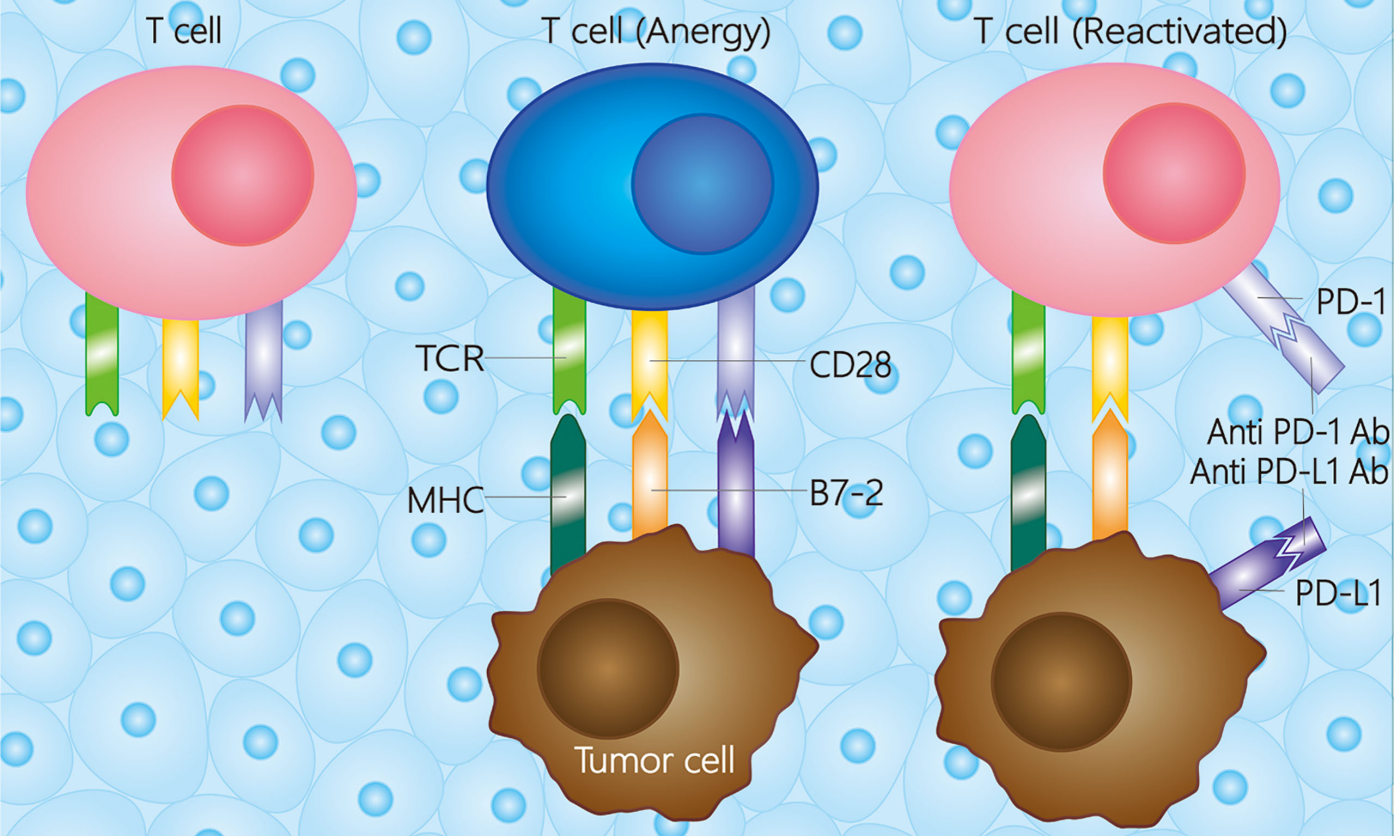
Mechanisms of PD-1/PD-L1 in the tumor microenvironment. Besides the first and second activation pathway between tumor cell and T cell, the T cell would be anergy when the PD-L1 combines with PD-1. While the T cell could be reactivated whether a PD-1 antibody or PD-L1 antibody combines with the receptor on T cell or tumor cell, respectively. The PD-1 antibody is referred to pembrolizumab, nivolumab, toripalimab, camrelizumab, et al., and the PD-L1 antibody is referred to atezolizumab, durvalumab, et al. TCR, T cell receptor; MHC, major histocompatibility complex; PD-1, programmed death receptor-1; PD-L1, programmed death ligand-1; Ab, antibody.

PD-1 is a cell-surface receptor expressed on peripheral tissue lymphocytes, such as T cells, natural killer cells, dendritic cells, and monocytes, that can bind to its ligand, PD-L1 (B7-H1 or CD274) or PD-L2 (B7-DC or CD273). PD-L1 can be expressed on tumor cells, dendritic cells, macrophages, T cells, or B cells through the immune microenvironment (28, 29). The combination of PD-1 and PD-L1 affects T-cell receptor (TCR) signaling, decreasing the threshold of apoptosis of T cells and reducing T-cell activity to facilitate tumor cells (29). The PD-1/PD-L1 pathway has also been found to regulate B cell activation; thus, PD-1 may be a vital checkpoint for T cell-dependent B cell activation and immunoglobulin (30). Previous research shows that the immune system can be re-stimulated by blocking PD-L1 expression *via* the MAPK pathway to induce an anti-tumor reaction (31), indicating that PD-1/PD-L1 monoclonal antibodies have the potential to treat malignancies (**Figure 1**).

Tumor cells highly express PD-L1 on their surface. Immunosuppression could be eliminated by blocking the PD-1/PD-L1 pathway to reactivate the immune system and achieve anti-tumor activity (12). While the individual heterogeneity of immune therapy is dependent on disparities in the tumor microenvironment (32), several studies have shown that tumor-associated macrophages (TAMs) and tumor-infiltrating lymphocytes (TILs) play an important role in tumor proliferation and invasion (33, 34). The inflammatory microenvironment characterized by abundant infiltration of T lymphocytes might indicate the promising efficacy of immunotherapy in cervical cancer (35). Tumors with inflamed immune profiles are superior to those with an immune-excluded or immune-desert profile in activating the immune system after immunotherapy (36). Tumor neoantigens produced by mutated genes enhance immunogenicity and might stimulate the immune system to identify and eliminate tumor cells more efficiently (37). Tumor microenvironment immune types (TMIT) have been classified into four categories, with cervical cancer specified as TIMT I with high expression of PD-L1 and CD8A/cytolytic activity (CYT), according to the classification criteria of TMIT by expression of PD-L1 and CD8A/CYT. Although there is no consensus on TMIT criteria, it is usually considered such patients may benefit from anti-PD-1/PD-L1 checkpoint blockade monotherapy (37).

### HPV infection and the immune-microenvironment of cervical cancer

HPV infection is highly associated with the malignant transformation of cervical intraepithelial neoplasia (CIN) and the development of cervical cancer. HPV16 and HPV18 are the predominant viral subtypes that promote malignant transformation of the cervix, accounting for 80% of all cases (38–40). The genes encoding E6 and E7 proteins in the HPV viral genome are suggested to promote the tumor suppressor gene p53 and retinoblastoma protein degradation, thus resulting in malignant transformation (41, 42). Under physiological conditions, most HPV infections can be obliterated by the immune system, whereas persistent HPV infection can lead to virus integration with the host cell genome. Several virus genes are missed in the process, including the E2 gene, which negatively transcribes and regulates E6 and E7 genes. This results in the persistent expression of E6 and E7 genes and ultimately leads to the malignant transformation of cervical cancer cells (43, 44).

With high correlations between HPV infection and cervical cancer, PD-L1 expression is reported to be related to HPV infection in cervical cancer. Compared with normal cervix tissue and other reproductive tumors, PD-L1 is significantly upregulated in atypical hyperplasia, CIN, or cervical squamous cell carcinoma (SCC) (12, 45–48). It has been postulated that the expression pattern of PD-L1 might serve as a predictive biomarker for HPV infection and malignant transformation of the cervix and also as a potential indicator for the elimination of HPV infection, even though the molecular mechanisms of how HPV infection induces elevated PD-L1 expression are still in the research phase (49). The expression of E6 and E7 virus genes is considered critical for upregulated PD-L1 expression (45, 50, 51); Mezache showed that several early open reading frames of these genes played a crucial role in the expression of PD-L1 in cervical cancer cells (12). Nevertheless, integrating HPV with PD-L1 gene fragments could lead to an enhanced PD-L1 allele and elevated PD-L1 expression (52).

Furthermore, HPV infection might elevate PD-1/PD-L1 expression in the immune microenvironment of cervical cancer and induce high cytotoxic T lymphocyte infiltration and abnormal cytotoxic T lymphocyte function (53). Yang reported that the expression levels of PD-1 and PD-L1 were elevated in T cells and DC cells of the cervix and were also correlated with CIN classification among those with HPV-positive cervical cancer (47, 54). DC cells have a strong antigen-presenting role and deliver both co-stimulatory and co-inhibitory signals to T cells. Thus, blocking the PD-1/PD-L1 pathway could inhibit antigen-presenting, leading to downregulation of the first signal and suppression of T-cell activation. Moreover, Mezache found that PD-L1 was elevated in monocytes in CIN or SCC compared to the normal cervix (12). Also, PD-1/PD-L1 pathway activation may negatively regulate the Th1 cytokine family (e.g., IFN-γ, IL-12) to promote immune response and positively regulate the Th2 cytokine family (e.g., IL-10, TGF-β) to suppress the immune response, thus leading to immune suppression of the tumor microenvironment (12).

In conclusion, the immune-suppressive PD-1/PD-L1 pathway is upregulated in HPV-associated CIN, which negatively regulates the immune response to HPV mediated by cervical cells, ultimately resulting in the malignant transformation of HPV-associated CIN. Theoretically, ICIs targeting the PD-1/PD-L1 pathway are a promising approach for HPV-associated cervical cancer.

### Expression pattern of PD-1/PD-L1 in cervical cancer and its relationship with clinical and histopathological characteristics

Cervical cancer is reported to express PD-1 or PD-L1, which might be more common in patients with certain clinical characteristics. The PD-L1 expression was quantified by the percentage of partial or complete membrane staining on the tumor cells of any intensity, which was also termed as tumor proportion score (TPS). The combined positive score (CPS) was another indicator, and referred to as the ratio of whole PD-L1 staining cells (including tumor cells, lymphocytes, and macrophages) to whole tumor cells and multiplied by 100 (55). In Arguello’s research, 63.1% of 84 cervical cancer samples were reported to have positive PD-1 expression without a positive cut-off value being specified (56). Meng reported that 70.1% of patients showed positive PD-L1 expression, while 68.0% showed positive PD-1 expression in cervical cancer; both of these were more commonly observed in patients with advanced-stage carcinoma, lymph node metastasis, vascular invasion, HPV infection, or previous history of neoadjuvant chemotherapy (46). Feng noted that 59.1%, 47.0%, and 60.6% of patients were found with positive PD-L1 expression in cancer cells, positive PD-L1 expression in TILs, and positive PD-1 expression in TILs with a cut-off value of 10%, respectively. Individuals with higher parity and cases of abortion were found to be associated with higher expression of PD-L1 in cancer cells, while those aged >55 years had the lowest PD-L1 expression in TILs, which may indicate an unfavourable therapeutic effect of anti-PD-1/PD-L1 drugs in elderly patients (57). Grochot reported that PD-L1 expression was observed in 32.2% of 59 cervical cancer samples, among which 8.5% was higher than 50% when a positive expression was detected on the cell membrane. Meanwhile, positive PD-L1 expression of infiltrated lymphocytes was detected in 27.1% of cervical cancer samples (58).

Apart from clinical characteristics, some histopathological features of cervical cancer are also possibly related to the expression pattern of PD-L1, although some of the findings from different studies were inconsistent with each other (58). For example, higher expression of PD-L1 was observed in SCC than in adenocarcinoma (AC; 54% versus 14%) with a cut-off value of 5%, and SCC of >15 mm infiltration depth was associated with comparatively lower expression of PD-L1 (*P*=0.025) (59, 60). Saglam noted that poorly differentiated SCC was related to higher PD-L1 expression than moderately differentiated malignancy (61). Inconsistently, in Reddy’s study, well and moderately differentiated malignancies were associated with higher PD-L1 expression in SCC than poorly differentiated malignancies. However, this discrepancy might be explained by unbalanced samples regarding cellular differentiation (59). In young patients, high expression of PD-L1 was associated with poorly differentiated SCC, which may be explained by thymic involution and lower amounts of T-cell progenitors resulting in lower naive T-cell production (61). Regarding the PD-L1 expression pattern of immune cells in the tumor microenvironment, immune cells with PD-L1 expression were more likely to surround metastatic tumors either in SCC (*P*=0.001) or AC (*P*=0.041) (60) when compared with paired primary tumors. Moreover, higher PD-L1 expression on tumor cells was observed in CIN than in SCC (95% versus 51%). However, the situation was reversed for mononuclear cells, where PD-L1 expression was less common in CIN than in SCC (61% versus 80%) (12). Furthermore, some studies suggested that lymph node metastasis and vascular invasion were correlated with positive expression of PD-L1, but this was overruled by Saglam’s study (46, 61).

In summary, patients with HPV infection (12, 46, 60), SCC (60), advanced stage (46, 60), large tumor size (61), poorly differentiated tumors (61), metastatic tumors (60), history of multiple parity and abortion (57), and a previous history of receiving chemotherapy (46) tend to have a higher expression of PD-L1, though more evidence is still needed. Nevertheless, the PD-L1 immunohistochemistry (IHC) assays could be confounded by various factors, including variable detection antibodies, sample preparation, processing variability, biopsy origins, intratumoral heterogeneity, evaluation assays (TPS or CPS), and the effect of chemotherapy and radiotherapy to induce PD-L1 expression (55). Accordingly, the PD-L1 expression evaluation criteria were supposed to be standardized and sufficiently reproducible. An in-depth knowledge of PD-L1 IHC assays could help to instruct the clinical practice, and the accurate evaluation of expression level might help to reflect the lowdown inside the tumor.

FDA approved four PD-L1 IHC assays (22C3, 28-8, SP263, and SP142) in clinical practice. The 22C3, 28-8, and SP142 assays was companion tests for pembrolizumab, nivolumab, and atezolizumab, and the SP263 assay was for pembrolizumab, nivolumab, and durvalumab. Among the four assays, 22C3 was the most widely used previously due to the first extensive use of Pembrolizumab. A high concordance among the 22C3, 28-8, and SP263 assays was noted, while SP263 seemed to show higher sensitivity than 28-8 and 22C3 (55). In this way, the discrepancy in the PD-L1 assay sensitivity and precision might compromise the accuracy of PD-L1 detection in clinical practice and clinical research, which should also be noted by the clinicians in further studies of cervical cancer.

### The association between PD-L1 expression and prognosis

In the era of conventional anti-tumor treatment such as surgery, chemotherapy, and radiotherapy, there was no clear evidence indicating the association between PD-L1 expression and survival. However, there was a trend that high PD-L1 expression might be related to a worse prognosis in advanced or metastatic cervical cancer patients. A retrospective study included 59 patients, of which 86.4% were SCC and 18.7% were clinical stage IV, indicating a shorter progression-free survival (PFS) in the PD-L1 positive-expressed population compared to the PD-L1 negative-expressed population, but with no statistical difference (11.5 versus 24.3 months; P=0.263) (58). Another retrospective study included 120 patients with locally advanced cervical cancer (88% were SCC), and they all received radical radiotherapy and platinum-based chemotherapy, among which 95.7%, 87.9%, and 73.3% were detected as positive PD-L1 expression with the cut-off value settings of ≥0%, ≥1%, and ≥5% for positivity, respectively. The conclusion was drawn that PD-L1 expression was not associated with OS and PFS, even though there was a tendency for high PD-L1 expression to be correlated with worse survival (58). Consistently, Karim’s study suggested that PD-L1 expression on tumor cells might have no direct influence on survival (58), while patients with both positive PD-L1 expression on tumor cells and infiltrating Tregs might have better survival (62). Moreover, for patients with positive PD-L1 expression, PD-L1 expressed at the tumor margin (tumor-stromal interface) rather than diffuse expression was more favourable for survival. PD-L1-positive TAM was considered poor disease-specific survival and more likely to be detected in SCC than AC (53% versus 12%, P<0.001) (60).

In conclusion, the positive expression of PD-L1 on tumor cells could not be neglected in cervical cancer. For those with HPV infection (12, 46, 60), SCC (60), advanced stage (46, 60), large tumor size (61), poorly differentiated tumors (61), metastatic tumors (60), history of multiple childbearing and abortion (57), and previous history of receiving chemotherapy (46), a high expression of PD-L1 might be observed, thus suggestion potential beneficiaries for anti-PD-1/PD-L1 treatment. Currently, no evidence has demonstrated a correlation between the expression level of PD-L1 and the prognosis of metastatic cervical cancer with conventional chemoradiotherapy. However, with the application of ICIs among patients with cervical cancer, some preliminary findings have shown the efficacy and safety of PD-1/PD-L1 inhibitors in selected patients, suggesting that PD-L1 expression might be one of the prognostic factors for the efficacy of immunotherapy. Nevertheless, some unspecified criteria may lead to a non-negligible impact on the clinical practice of PD-1/PD-L1-associated treatment and prognosis prediction. Firstly, the expression of PD-L1 in tumor tissues depends on the tumoral biological characteristics and intratumoral heterogeneity. There may be an inductive and dynamic expression of PD-L1, with different distribution characteristics in tumor cells and immune cells. Secondly, PD-L1 detection methods, including different detection antibodies, different detection platforms and methodologies, different criteria for cut-off values, and bias of different pathologists or technicians in determining specimens, may lead to inconsistent PD-L1 detection results. Furthermore, various sample sites (primary versus metastatic lesions) and various specimen qualities may affect PD-L1 expression levels (63–65). Further standardization of PD-L1 assay techniques and interpretation criteria are needed to provide stronger support and evidence for the clinical practice of PD-1/PD-L1 inhibitors in cervical cancer.

## Clinical evidences for PD-1/PD-L1 ICIs in cervical cancer

The FDA first approved two PD-1 ICIs (nivolumab and pembrolizumab) for unresectable or metastatic melanoma, NSCLC, and renal cell carcinoma in September 2014. With the promising efficacy and broad utilization of immunotherapy in treating melanoma and NSCLC, it is still under exploration for cervical cancer, with several clinical studies revealing the potential benefits. Apart from anti-PD-1 antibodies, anti-PD-L1 monoclonal antibodies (atezolizumab) also show potential efficacy in bladder cancer and NSCLC. Moreover, nivolumab and pembrolizumab have also been effective in early-stage (66) and advanced carcinoma (67, 68). To date, 15 clinical trials have reported their findings for cervical cancer (**Table 1**), while another two clinical trials (78, 79) are ongoing for advanced or metastatic cervical cancer. Among the reported clinical trials, two focused on pembrolizumab (16, 17, 69–71), three on nivolumab (18–20), one on sintilimab (72), two on balstilimab (73, 74), one on camrelizumab (75), two on cemiplimab (76, 77), one on atezolizumab (21). Afterwards, we would review the outcomes of pembrolizumab, nivolumab, and atezolizumab in detail and also prospect the ongoing clinical trials.

**Table 1:** Outcomes of the reported clinical trials of immune checkpoint inhibitors in cervical cancer.

Trial	Author,year	Phase	Masking	Study population	Drug	Dose^a^(Dosing Interval)	Primary endpoint	Secondary endpoint	
Keynote 028	Frenel, 2017 (16)	Ib	Open-label	Locally advanced/metastatic PD-L1–positive cervical cancer, which had progressed after standard therapy	Pembrolizumab	10 mg/kg (2 weeks)	ORR	AE, Tolerability, PFS, OS, DOR	
Keynote 158	Chung, 2019 (17)	II	Open-label	Advanced cervical cancer progressed after standard therapy	Pembrolizumab	200 mg (3 weeks)	ORR	DOR, PFS, OS	
/	Duska, 2020 (69)	II	Open-label	Stage IB-IVA cervical cancer	Pembrolizumab	200 mg (3 weeks)	AE, DLTs	MRR, PFS, OS	
/	Youn, 2020 (70)	II	Open-label	Recurrent or advanced HPV-positive cervical cancer	Pembrolizumab (+GX-188E)	200 mg (3 weeks)	ORR	AE, BOR, TBR, DOR, OS, PFS	
/	Colombo, 2021 (71)	III	Double-blind	Persistent, recurrent, or metastatic cervical cancer	Pembrolizumab	200 mg (3 weeks)	OS, PFS	ORR, DOR, 1-yr PFS rate, AE	
CheckMate 358	Naumann, 2019 (18)	I, II	Open-label	Recurrent/metastatic cervical, vaginal, or vulvar cancers	Nivolumab	240 mg (2 weeks)	ORR	DOR, PFS, OS	
NRG- GY002	Santin, 2020 (19)	II	Open-label	Persistent/recurrent cervical cancer, failure of prior systemic therapy	Nivolumab	3 mg/kg (2 weeks)	ORR, AE	PFS, OS	
/	Tamura, 2019 (20)	II	Open-label	Advanced/recurrent uterine cervical cancer, uterine corpus cancer, soft tissue sarcoma	Nivolumab	240 mg (2 weeks)	ORR	BOR, DCR, OS, PFS, DOR, MPC	
/	Xu, 2022 (72)	II	Open-label	PD-L1–positive recurrent or metastatic cervical cancer	Sintilimab	200 mg (3 weeks)	ORR	PFS, OS, DCR	
/	O’Malley, 2021 (73)	II	Open-label	Previously-treated, recurrent/metastatic cervical cancer	Balstilimab	3 mg/kg (2 weeks)	ORR	DOR, DCR	
/	O’Malley, 2021 (74)	II	Open-label	Recurrent and/or metastatic cervical cancer who relapsed after prior platinum-based therapy	Balstilimab (+Zalifrelimab)	3 mg/kg (2 weeks)	ORR	DOR, AE, PFS, OS	
CLAP	Lan, 2020 (75)	II	Open-label	Advanced cervical cancer that progressed after at least one line of systemic therapy	Camrelizumab (+Apatinib)	200 mg (2 weeks)	ORR	PFS, OS, DOR, DCR, AE	
/	Rischin, 2020 (76)	I	Open-label	Recurrent or metastatic cervical cancer	Cemiplimab	3 mg/kg (2 weeks)	AE	ORR, DCR, TTR, DOR	
/	Tewari, 2022 (77)	III	Open-label	Recurrent or metastatic cervical carcinoma that progressed after platinum-containing therapy	Cemiplimab	350 mg (3 weeks)	OS	PFS, AE	
/	Friedman, 2020 (21)	II	Open-label	Advanced cervical cancer	Atezolizumab	15 mg/kg (3 weeks)	ORR	DCR, PFS, OS, safety	
Author, year		Evaluable patients (n)	Age (years, range)	ORR (95%CI)	Median PFS (month, 95%CI)	Median OS (month, 95%CI)	Adverse Effect	
							Any grade (n)	Grade≥3 (n)
Frenel, 2017		24	42 (26-62)	17% (5%-37%)	2 (2-3)	11 (4-15)	75% (18)	20.8% (5)
Chung, 2019		98	46 (24-75)	12.2% (6.5%-20.4%)	2.1 (2.0-2.2)	9.4 (7.7-13.1)	65.3% (64)	12.2% (12)
Duska, 2020		88	NA	NA	NA	NA	88% (46)^d^	65% (34)^e^
Youn, 2020		36/26^b^	51	42% (23%-63%)	4.9 (2.1–6.7)	10.2 (6.6-16.7)	44% (16)	11% (4)
Colombo, 2021		617	51 (25–82)^c^	NA	10.4 (9.1-12.1)	24.4	99.3% (305)	81.8% (251)
Naumann, 2019		19	51 (28-75)	26.3% (9.1%-51.2%)	5.1 (1.9-9.1)	21.9 (15.1-NR)	63.2% (12)	21.1% (4)
Santin, 2020		25	45	4%	3.5 (90% CI, 1.9–5.1)	14.5 (90% CI, 8.3–26.8)	84% (21)	32% (8)
Tamura, 2019		20	50 (32-68)	25% (80%CI, 13%-41%)	5.6 (80% CI, 2.8‐7.1)	NR	65% (13)	20% (4)
Xu, 2022		42	53 (36-67)	54.8% (38.7%-70.2%)	9.4 (8.0-14.6)	NR	85.8% (36)	16.7% (7)
O’Malley, 2021		161	53 (25-81)	15% (10.0%-21.8%)	NA	NA	71.4% (115)	11.8% (19)
O’Malley, 2021		155	50 (24-76)	25.6% (18.8%-33.9%)	2.7 (1.5-3.7)	12.8 (8.8-17.6)	70.0% (110)	20.0% (31)
Lan, 2020		45	51 (33-67)	55.6% (40.0%-70.4%)	8.8 (5.6-NE)	NR	95.6% (43)	71.1% (32)
Rischin, 2020		20	55 (31–76)^f^	10.0% (0.3%–44.5%)	1.9 (1.0–9.0)	10.3 (2.1-NE)	90% (9)	40% (4)
Tewari, 2022 (77)		608	51 (22-87)	16.4% (12.5%-21.1%)	2.8 (2.6-3.9)	12.0 (10.3-13.5)	88.3% (265)	45.0% (135)
Friedman, 2020		11	48 (31-55)	60%	2.9 (1.8-6)	8.9 (3.4-21.9)	NA	36.4% (4)

^a^Only the doses of the study PD-1/PD-L1 inhibitors were presented, and the doses of other drugs (e.g., vaccine, CTLA-4 antibody) were not shown.

^b^Youn’s study assessed the safety outcomes in 36 patients and efficacy outcomes in 26 patients.

^c^The age of Colombo’s study merely presented the median age and the range of the experimental group.

^d^The adverse events of all grades merely presented the grade 2 or higher adverse events of the 52 patients who completed treatment and assessment among the planned 88 patients.

^e^The adverse events of grade 3 or higher merely presented the severe adverse events of the 52 patients who completed treatment and assessment.

^f^The age of Rischin’s study merely presented the median age and the range of the cemiplimab monotherapy group.

AE, adverse event; BOR, best overall response; CI, confidence interval; DCR, disease control rate; DOR, duration of response; HPV, human papillomavirus; IC, immunologic changes; MPC, the maximum percentage change in tumor size and percentage change over time; MRR, the metabolic response rate on positron emission tomography-computed tomography imaging; NE, not evaluable; NR, not reached; ORR, objective response rate; OS, overall survival rate; PD-L1, programmed death receptor-1 ligand; PFS, progression-free survival; TBR, time to best response; TTR, time to response; yr, year.

### Pembrolizumab

Phase Ib KEYNOTE-028 study was a single-arm and basket trial using pembrolizumab 10 mg/kg every two weeks. The enrollment of subjects included 24 patients with pretreated locally advanced or metastatic cervical cancer and positive PD-L1 expression (cut-off value, 1%), among which 96% were SCC. The objective response rate (ORR) was 17% (95% confidential interval [CI], 5-37%), median PFS (mPFS) was two months (95%CI, 2-3 months), mOS was 11 months (95% CI, 4-15 months), and the duration of response (DOR) was 5.4 months (range, 4.1-7.5 months). In the research, 75% of the cases experienced AEs, with only rash (n=5; 21%) and pyrexia (n=4; 17%) occurring in 10% of patients. Five patients experienced grade 3 treatment-related AEs. Six patients experienced immune-mediated AEs including rash (n=2; grade 3), colitis (n=1; grade 3), Guillain-Barré syndrome (n=1; grade 3), hyperthyroidism (n=1; grade 2), and hypothyroidism (n=1; grade 2). Pembrolizumab was therefore considered tolerable and with persistent anti-tumor activity in patients with PD-L1-positive advanced cervical cancer (16).

The Phase II KEYNOTE-158 study was a basket-designed and open-label study of pembrolizumab (200mg every three weeks), including 98 patients with pretreated locally advanced or metastatic cervical cancer. The ORR, mPFS, and mOS were 12.2% (95%CI, 6.5–20.4%), 2.1 months (95%CI, 2.0–2.2 months), and 9.4 months (95%CI, 7.7–13.1 months), respectively. The actual DOR was not reached, ranging from ≥3.7 to ≥18.6 months. In total, 65.3% of the patients experienced AEs, and 12.2% experienced severe AEs (grade≥3), the most common were increased alanine aminotransferase (3.1%) and increased aspartate aminotransferase (2.0%). PD-L1-positive patients (CPS≥1) showed a higher ORR of 14.6%, compared to no response observed in the PD-L1-negative patients. Pembrolizumab showed a favourable efficacy that the mOS was 9.4 months in the total population and 11.0 months in PD-L1-positive patients (17).

Duska conducted a randomized, phase 2, open-label study on pembrolizumab (200mg every three weeks) for stage IB-IVA cervical cancer, and it compared the safety and efficacy between pembrolizumab after pelvic chemoradiotherapy and pembrolizumab during pelvic chemoradiotherapy. Duska first reported the safety outcomes of the two arms, and a total of 52 patients (88 patients planned to be enrolled) had completed the treatment and safety assessment. Overall, 88% of the involved patients experienced AEs (grade≥2), and 65% experienced severe AEs (grade 3 and 4), while no grade 5 AE was reported. These findings suggested the feasibility of the concurrent or sequential treatment of pembrolizumab and pelvic chemoradiotherapy (69).

Youn conducted a single-arm, phase 2 trial on pembrolizumab and GX-188E (DNA vaccine) for those with HPV-16/18-positive, recurrent or advanced, inoperable cervical cancer, and it showed manageable AEs and satisfactory antitumor activities. A total of 36 patients received the study treatment (≥1 dose), of which 26 received at least 45 days of the study treatment and completed the efficacy assessment. In total, 11 (42%) patients and 15 (58%) patients achieved overall response and disease control, respectively. As for the safety outcomes, 16 (44%) experienced AEs, and four experienced severe AEs. Among the 23 response-evaluable patients, the positive T-cell response induced by the GX-188E vaccine could be observed in 18 patients (70).

Colombo conducted the first phase III, double-blind trial on pembrolizumab plus chemotherapy with or without bevacizumab for persistent, recurrent, or metastatic cervical cancer. In summary, 617 patients were included, of which 548 had PD-L1 combined positive score (CPS) ≥1, 317 had PD-L1 CPS≥10, 56.4% had chemoradiotherapy with or without surgery, and 63.6% and 62.5% received bevacizumab in the pembrolizumab and placebo groups, respectively. In the pembrolizumab group of the intention-to-treat population, the median PFS and OS were 10.4 months and 24.4 months, respectively. The 24-month OS rate estimate was 50.4% versus 40.4% for the pembrolizumab and placebo group, respectively, and the hazard ratio (HR) was 0.67 (95% CI, 0.54-0.84). Among those with PD-L1 CPS≥1, the overall response was significantly higher in the Pembroliaumab group (68.1% versus 50.2%). Severe AEs could be observed in 81.8% and 75.1% of the pembrolizumab and placebo groups, respectively. As for the patient-reported outcomes, the pembrolizumab group showed a longer time of deterioration in the EuroQol Group 5-Dimension 5-Level questionnaire compared to the placebo group (58.2% versus 44.8%; HR=0.75; 95% CI, 0.58) (71).

### Nivolumab

The CheckMate 358 trial, a phase I/II, single-arm, and open-label study, included 24 patients with recurrent/metastatic cervical, vaginal, or vulvar cancers with 240 mg nivolumab prescribed every two weeks. In this research, 19 patients were diagnosed with cervical cancer, all of which previously received at least one-line treatment (PD-L1 positivity expression, CPS≥1). The ORR and disease control rate (DCR) was 26.3% (95%CI, 9.1-51.2%) and 68.4% (95%CI, 43.4-87.4%) in patients with cervical cancer, respectively. The mPFS, mOS, and 12-month OS rate were 5.1 months (95%CI, 1.9-9.1 months), 21.9 months (95% CI, 15.1 months-NR), and 77.5% (95%CI, 50.5-91.0%), respectively. The occurrence rate of AE was 63.2% (12/19), with the most common treatment-related AEs being diarrhea (4/19, 21.1%); severe AE occurrence was 15.8% (3/19), including diarrhea, pneumonitis, and hepatocellular injury. One patient discontinued the treatment owing to grade 3 pneumonitis (18).

Tamura’s study was a phase II and open-label clinical trial, assessing nivolumab (240 mg every two weeks) in 20 patients with advanced/recurrent cervical cancers who received at least one chemotherapy regimen [PD-L1 positivity expression, tumor proportion score (TPS) ≥1%]. The ORR was 25% (80%CI, 13-41%), mPFS was 5.6 months (80%CI, 2.8-7.1 months), mOS was not reached (NR), and the 6-month OS rate was 84% (80%CI, 70-92%). In a subgroup analysis, ORR was 33% in the PD-L1-positive subgroup versus 0% in the PD-L1-negative subgroup. The AE occurrence was 65% (13/20), with the occurrence rate of severe AE ≥ grade 3 as 20% (4/20), including increased lipase, maculopapular rash, increased γ‐glutamyl transferase, and spondylitis. Although there was sudden death from cervical cancer, it was not viewed as correlated with immune reaction or nivolumab (20).

In Santin’s study, a phase II study of nivolumab was conducted, enrolling 25 patients with persistent, recurrent, or metastatic cervical cancer who had received at least one prior chemotherapy. The positivity of PD-L1 expression (CPS≥1) was identified in tumor cells in 63.6% of the patients and immune cells in 72.7%. In this trial, the ORR and DCR were 4% and 40%, respectively. The mPFS was 3.5 months (90%CI, 1.9-5.1 months), mOS was 14.5 months (90%CI, 8.3-26.8 months), and the estimated 6-month PFS and OS rate was 16% and 78.4%, respectively. In the research, 21 (84%) patients suffered from AEs, while 6 (32%) experienced severe AEs, including increased blood bilirubin (grade 4) and increased serum amylase (grade 4) (19).

### Atezolizumab

Friedman reported a phase II, open-label, multicenter study that evaluated the safety and efficacy of atezolizumab in combination with bevacizumab, in which 11 patients with advanced cervical cancer were included (21). In the total evaluable population (n=10), zero patients achieved an objective response, resulting in a confirmed ORR of 0%. The DCR was 60%, all of which was stable disease. The mPFS was 2.9 months (95%CI, 1.8-6 months), and median OS was 8.9 months (95%CI, 3.4-21.9 months). The occurrence rate of severe AE was 36.4%, and no treatment-related death was reported, but two patients discontinued treatment owing to grade 3 neurologic events. No significant survival benefit was concluded concerning tumor PD-L1 expression (*P*=0.663), tumor CD8^+^ T cell infiltration (*P*=0.868), or stromal PD-L1 expression (*P*=0.867). Of note, there were two patients who achieved an unconfirmed PR who had PD-L1 CPS>1 (1.44 and 7.07, respectively). Therefore, this trial concluded that the combination of bevacizumab and atezolizumab did not meet the predefined efficacy endpoint, as the addition of bevacizumab to PD-L1 blockade did not appear to enhance the ORR in cervical cancer.

### Cemiplimab

Rischin investigated the safety and anti-tumor activity of cemiplimab monotherapy with or without hypo-fractionated radiotherapy for recurrent or metastatic cervical cancer from a non-randomized phase I expansion cohort, in which a total of 20 patients were included (76). The median follow-up for the monotherapy cohort was 5.6 months (range, 0.8–16.2), and it was 3.76 months (range, 0.7–8.1) for the combination cohort. The DCR was 40.0% (95%CI, 12.2–73.8%) and 60.0% (95%CI, 26.2–87.8%), and DOR was 11.2 and 6.4 months for the monotherapy and combination cohort, respectively. The AE could be observed in nearly all the enrolled patients, and the severe AEs were 40% for either of the cohort. It reminded us of the controllable safety and preliminary anti-tumor activity, and the favored outcomes were consistent with the results from pan-cancer (80).

Tewari revealed the first multicenter, phase III trial to investigate the efficacy and safety of cemiplimab in the patients with recurrent or metastatic cervical cancer progressed after platinum-based chemotherapy (77). The survival outcome was superior in cemiplimab to that of chemotherapy (mOS, 12.0 vs. 8.5 months), and the ORR was also favored in cemiplimab (16.4% vs. 6.3%). The severe AEs was less in the cemiplimab cohort than the chemotherapy cohort (45.0% vs. 53.4%). Positive PD-L1 expression (≥1%) was much more common in the SCC than AC (70.7% vs. 32.6%). As stratified by the PD-L1 expression, the clinical outcomes were superior in the PD-L1 expression ≥1% than <1% for those with cemiplimab (ORR, 18% vs. 11%; mOS, 13.9 vs. 6.7 months). The research reminded us of the potential role of cemiplimab in the PD-L1-negative patients and those with recurrent diseases.

### Ongoing clinical trials

Previous research concerning mechanisms and clinical trials suggests the potential safety and efficacy of ICIs in advanced or metastatic cervical cancer. Compared with traditional chemotherapy or radiotherapy, immune therapy may further improve survival and prognosis in such patients. Besides, previous trials have initial evidence of the potential of ICIs in recurrent/metastatic cervical cancer, and more trials are ongoing.

The PRIMMO trial is ongoing and aims to explore the efficacy of pembrolizumab combined with chemoradiotherapy in the hope of mediating anti-tumor immunity *via* changing the tumor microenvironment. Immune-related biomarkers are also being investigated in this trial (78). The Phase I DURVIT trial was designed to demonstrate whether intratumor injection of durvalumab could control the early metastatic spread of cervical cancer cells through the lymph node drainage area in the hope of delaying or preventing disease recurrence (79). Currently, no studies have systematically compared efficacy and safety among anti-PD-1 drugs and anti-PD-L1 drugs in cervical cancer.

By June 2022, there were 153 clinical trials involving pembrolizumab, atezolizumab, durvalumab, nivolumab, and other PD-1/PD-L1 monoclonal antibodies in cervical cancer, including 44 for pembrolizumab, 14 for nivolumab, 12 for camrelizumab, 16 for atezolizumab, and 11 for durvalumab (**e-Table 1**). These trials include patients with advanced, recurrent, or metastatic cancer, ranging from first-line to multi-line treatment. The clinical trials are mostly distributed in phase I and phase II, and the only ten clinical trials are phase III (NCT04157985, NCT03912415, NCT04806945, NCT04864782, NCT04943627, NCT03635567, NCT03755739, NCT04221945, NCT03556839, and NCT03830866). Current clinical trials have mainly been ongoing since 2015, and the clinical trial of Durvalumab combined with Tremelimumab (NCT01975831) was initiated in 2013.

Most trials concerning pembrolizumab or Atezolizumab focused on monotherapy, while the trials concerning Durvalumab were commonly combined with tremelimumab (anti-CTLA-4 antibody), and Nivolumab was widely combined with ipilimumab. Vaccines were also combined with ICIs to investigate the potential clinical efficacy (NCT03073525, NCT03444376, NCT02291055, and NCT03439085). The QUILT-3.055 trial (NCT03228667) compared the clinical efficacy of pembrolizumab in combination with ALT-803 (IL-15 hyperagonist) in advanced cancer. The National Cancer Institute (NCI) conducted a MATCH Screening Trial (NCT02465060) of 6452 patients with solid tumors or lymphoma who progressed after first-line standard treatment, which was the largest clinical trial on the efficacy in patients with different malignancies. The ATEZOLACC trial (NCT03612791) investigated the efficacy of the addition of Atezolizumab to standard chemoradiotherapy, which might be the main direction of future studies that place immunotherapy earlier in the therapeutic strategy. There is a tendency for different types of immunotherapy, such as an anti-CTLA4 monoclonal antibody, vaccine, or cytokine agonist, to be applied together with PD-1/PD-L1 inhibitors. Furthermore, triple combination treatment was another potential strategy for exploration, such as a PD-L1 inhibitor plus chemotherapy combined with an antiangiogenic agent (e.g. bevacizumab).

## Conclusions

As a common female reproductive malignancy, cervical cancer is associated with high-risk HPV infections. The inflammatory tumor microenvironment led by chronic HPV infection might be available for immunotherapy in cervical cancer. In this paper, we systematically described the characteristics of the tumor microenvironment and the expression pattern of PD-L1 in cervical cancer. We also explored the clinical and histopathological features of patients with positive PD-L1 expression, including SCC, poor differentiation, advanced stage, large tumor size, concomitant HPV infection, history of multiple parity and abortion, and previous history of receiving chemotherapy, who were also potential beneficiaries from PD-1/PD-L1 inhibitors.

With the increasing application of immunotherapy in malignancies, many findings with directive significance have been implied for PD-1/-PD-L1 inhibitors in cervical cancer. PD-1 inhibitors, including pembrolizumab and Nivolumab, have demonstrated initial efficacy and safety for advanced/metastatic cervical cancer as second-line monotherapy in several phase II clinical trials. Hence, a series of immunotherapy clinical studies are flourishing, and ongoing clinical research has developed from small-scale phase II single-arm trials to phase III randomized controlled trials, from second-line and multi-line therapy to first-line therapy, and from monotherapy to combined treatment modalities, including immuno-chemo-antiangiogenic therapy, immuno-chemo-radiotherapy, dual immunotherapy, and immunotherapy combined with small-molecule multi-target tyrosine kinase inhibitors. Nevertheless, the exploration of novel immunotherapy targets is continuing. We look forward to unveiling more high-quality clinical data on a large scale in the future to illuminate the treatment of cervical cancer from bench to bed.

## Author contributions

WH and JL contributed to the conception of the review. WH and HC searched the literature. WH, JL, and KX drafted the manuscript. WH, JL, and CB contributed to the revision of the manuscript. All authors approved the final version of the manuscript.

## Funding

This work was supported by the Key Research Projects of Science and Technology Department Foundation of Sichuan Province (No.2017SZ0141).

## Acknowledgments

The authors thank *Charlesworth* for reviewing and language polishing of the manuscript.

## Conflict of interest

The authors declare that the research was conducted in the absence of any commercial or financial relationships that could be construed as a potential conflict of interest.

The reviewer RT declared a shared affiliation with the authors to the handling editor at the time of review.

## Publisher’s note

All claims expressed in this article are solely those of the authors and do not necessarily represent those of their affiliated organizations, or those of the publisher, the editors and the reviewers. Any product that may be evaluated in this article, or claim that may be made by its manufacturer, is not guaranteed or endorsed by the publisher.

## Abbreviations

AC, adenocarcinoma; AEs, adverse events; CI, confidential interval; CIN, cervical intraepithelial neoplasia; CPS, combined positive score; CYT, cytolytic activity; DCR, disease control rate; DOR, duration of response; HPV, human papillomavirus; ICIs, immune checkpoint inhibitors; mOS, median overall survival; mPFS, median progression-free survival; NR, not reached; NSCLC, non-small cell lung cancer; ORR, objective response rate; OS, overall survival; PD-1, programmed death receptor-1; PD-L1, programmed death receptor-1 ligand; PFS, progression-free survival; SCC, squamous cell carcinoma; TAMs, tumor-associated macrophages; TCR, T-cell receptor; TILs, tumor-infiltrating lymphocytes; TMIT, tumor microenvironment immune types; TPS, tumor proportion score.
