# Supplementary material for: PD-1/PD-L1 inhibitors for advanced or metastatic cervical cancer: From bench to bed
Source: Front Oncol. 2022 Oct 14;12:849352. doi: 10.3389/fonc.2022.849352 (PMC9614140; doi:10.3389/fonc.2022.849352)
Supplement: Supplementary file 1 [file Table_1.docx]

**e-Table 1 Clinical trials of PD-1/PD-L1 immune checkpoint inhibitors in cervical cancer grouped by PD-1 antibody, PD-L1 antibody and combined PD-1/PD-L1 therapy**

| **Drug** | **ClinicalTrials.gov identifier** | **Phase** | **Study Start Date** | **Treatment stage** | **Combination** | **Study population** | **Population Scale** | **Primary Outcomes** | **Secondary Outcomes** |
| --- | --- | --- | --- | --- | --- | --- | --- | --- | --- |
| **PD-1 Antibody** |  |  |  |  |  |  |  |  |  |
| PD-1 Ab, NOS | NCT03589339 | I | January 16, 2019 | NOS | NBTXR3 (radioenhancer hafnium oxide nanoparticle) | Metastatic cancer | 60 | DLT, MTD, RP2D | ORR, SAE, PK |
| PD-1 Ab, NOS | NCT04116320 | I | November 21, 2019 | Multi-line | Focused Ultrasound Ablation, Imiquimod | Advanced solid tumor with measurable metastatic disease | 32 | AE, CD8+ T cell infiltration extent | CD8+ T cell infiltration extent after spot FUSA |
| PD-1 Ab, NOS | NCT04341883 | II | November 13, 2019 | Second-line | Albumin-bound paclitaxel | Recurrent cervical cancer | 55 | ORR | PFS, OS, DCR, DOR |
| PD-1 Ab, NOS | NCT04157985 | III | November 15, 2019 | NOS | / | Advanced solid tumors | 578 | TTNT, PFS | irAE, OS, BOR |
| PD-1 Ab, NOS | NCT05310331 | II | March 27, 2022 | Second-line | Donafenib, paclitaxel, and platinum | Recurrent cervical cancer | 20 | PFS | OS, ORR, DCR, AE, TEAE, SAE |
| AK104 | NCT04380805 | II | July 15, 2020 | Second-line | / | Recurrent or metastatic cervical cancer | 40 | ORR | ORR, DCR, DOR, PFS, AE et al. |
| AK104 | NCT04868708 | II | April 1, 2021 | First-line, second-line | Bevacizumab, Paclitaxel, Cisplatin/Carboplatin | Recurrent or metastatic cervical cancer | 60 | AE | ORR, DOR, DCR, PFS, OS et al. |
| AK104 | NCT05227651 | II | March 30, 2022  (estimated) | Neoadjuvant | / | IB2-IIA2 treatment-naïve cervical squamous cell carcinoma | 30 | AE, MPR | R0 resection rate, pCR, ORR |
| AK105 | NCT05137171 | II | March 1, 2022 | Multi-line | Anlotinib | Persistent, recurrent, and metastatic cervical cancer | 36 | ORR | PFS, OS |
| BCD-100 | NCT03912402 | II | December 25, 2018 | First-line | Bevacizumab | Metastatic, recurrent, or persistent cervical cancer | 49 | ORR | PFS, OS |
| BCD-100 | NCT03912415 | III | October 1, 2019 | First-line | Bevacizumab, Paclitaxel, Cisplatin/Carboplatin | Metastatic cervical cancer | 316 | OS | PFS, ORR, DCR, TTR, DOR |
| GB226 | NCT03808857 | II | May 23, 2019 | Multi-line | / | Recurrent or metastatic cervical cancer | 80 | ORR | DCR, TTR, DOR, PFS, OS |
| GLS-010 | NCT03972722 | II | May 15, 2019 | Multi-line | / | Recurrent or metastatic cervical cancer | 89 | ORR | PFS, DCR, DOR, OS, TTR |
| HLX10 | NCT04150575 | II | March 10, 2020 | Multi-line | Albumin-Bound Paclitaxel | Advanced cervical cancer | 143 | ORR | ORR, PFS, OS |
| HLX10 | NCT04806945 | III | September 30, 2022  (estimated) | First-line | Paclitaxel, cisplatin, carboplatin | Persistent, recurrent, or metastatic cervical cancer | 482 | PFS, OS | / |
| MGD013 | NCT03219268 | I | August 18, 2017 | Multi-line | Margetuximab | Unresectable or metastatic neoplasms | 353 | AE, SAE, MTD | PK, IG, PD, TEAE, OS |
| OC001 | NCT04260802 | I/II | October 6, 2020 | NOS | / | Locally advanced or metastatic cancers | 80 | DLT, SAE | PK, ORR, PFS, DOR, TTR, DCR, OS |
| QL1604 | NCT04864782 | II/III | September 23, 2020 | First-line | Paclitaxel, cisplatin, carboplatin | Stage IVB, recurrent, or metastatic cervical cancer | 458 | AE, SAE, TEAE, ORR, PFS | OS, ORR, DOR, TTP et al. |
| SG001 | NCT03852823 | I | May 23, 2019 | Multi-line | / | Advanced tumours | 192 | DLT, ORR | PK, ORR, DOR, DCR, TTP, PFS, OS |
| SG001 | NCT04886700 | II | July 31, 2021 | Multi-line | / | Relapsed or metastatic uterine cervical cancer | 104 | ORR | ORR, DOR, DCR, PFS, OS, TTR, TEAE |
| SL-279252 | NCT03894618 | I | March 26, 2019 | Multi-line | / | Advanced solid tumors or lymphomas | 87 | TEAE, MTD | ORR, IG, PK, PD |
| Sym021 | NCT04672434 | I | November 19, 2020 | Multi-line | Sym024 (anti-CD73) | Advanced solid tumor malignancies (including cervical cancer) | 100 | AE, SAE, MTD | ORR, TTP, PD, PK |
| TSR-042 | NCT03833479 | II | June 28, 2019 | Multi-line | / | High-risk locally advanced cervical cancer | 132 | PFS | AE, OS, PROs of QOL/fatigue/pain |
| XmAb20717 | NCT03517488 | I | July 10, 2018 | NOS | / | Advanced solid tumor | 154 | TEAE |  |
| AGEN2034  (Balstilimab) | NCT03104699 | I/II | April 11, 2017 | NOS | / | Recurrent, unresectable, metastatic, or advanced cervical cancer | 211 | ORR | TEAE, ORR, DOR, DCR, PFS, OS |
| AGEN2034  (Balstilimab) | NCT03495882 | I/II | December 18, 2017 | NOS | AGEN1884 | Locally advanced, recurrent and/or metastatic solid tumors | 154 | ORR | TEAE, DOR, DCR, PFS, OS et al. |
| AGEN2034  (Balstilimab) | NCT03894215 | II | June 1, 2019 | Second-line | AGEN1884 | Metastatic, locally advanced, and/or unresectable cervical cancer | 200 | ORR | TEAE, DOR, TEAE et al. |
| Balstilimab | NCT04943627 | III | August 2, 2021 | Multi-line | Gemcitabine, irinotecan, pemetrexed, vinorelbine, or topotecan | Recurrent, persistent, or metastatic cervical cancer | 486 | OS | PFS, ORR |
| Balstilimab | NCT05033132 | II | September 28, 2021 | Second-line | Zalifrelimab | Advanced (recurrent, unresectable, or metastatic cervical cancer | 160 | AE, ORR | / |
| SHR-1210  (Camrelizumab) | NCT03816553 | II | January 19, 2019 | Multi-line | Apatinib | Metastatic, persistent, or recurrent cervical cancer | 49 | ORR | PFS, OS, DCR, DOR, AE |
| SHR-1210  (Camrelizumab) | NCT03827837 | II | January 23, 2019 | Second-line | Famitinib | Advanced urinary system/gynecological tumors | 265 | ORR | DOR, DCR, TTR, PFS, OS, AE et al. |
| Camrelizumab | NCT04188860 | II | December 6, 2019 | Multi-line | Albumin-bound paclitaxel | Recurrent or persistent advanced cervical cancer | 34 | ORR | PFS, OS, DCR, RD, AE |
| Camrelizumab | NCT04508686 | I | August 1, 2020 | Third-line | Capecitabine | Advanced cervical cancer | 20 | AE | PFS, OS, ORR, DCR, DOR |
| Camrelizumab | NCT04635956 | II | November 15, 2020 | NOS | Bevacizumab, paclitaxel, platinum, etoposide | Recurrent or advanced cervical neuroendocrine carcinomas | 20 | ORR | SAE, functions of liver/ kidney/myocardium/ adrenal gland, insulin resistance |
| SHR-1210  (Camrelizumab) | NCT04516616 | II | December 1, 2020 | First-line | Cisplatin, Albumin-bound paclitaxel | Locally advanced cervical cancer | 84 | ORR | PCR, DFS, OS |
| Camrelizumab | NCT04974827 | II | May 20, 2021 | First-line | Cisplatin or carboplatin | Patient with cervical cancer who had recurrence of the pelvic wall after surgery ± abdominal aortic lymph node metastasis | 46 | CRR | ORR, DCR, OS, DOR, PFS |
| Camrelizumab | NCT04974944 | II | July 30, 2021 | First-line | Apatinib, aclitaxel, cisplatin/ carboplatin, bevacizumab | Stage IVB, recurrent or persistent cervical squamous cell carcinoma | 172 | PFS | ORR, DOR, DCR, OS, AE, PROs |
| Camrelizumab | NCT05290935 | II | March 13, 2022 | Multi-line | Albumin-bound paclitaxel | Recurrent or persistent advanced cervical cancer | 122 | ORR | PFS, OS, DCR, AE |
| Camrelizumab | NCT05311566 | II | March 27, 2022 | First-line | Cisplatin, radiotherapy | IB2-IIIB cervical cancer | 92 | OS | PFS, ORR, AE |
| Camrelizumab | NCT05234905 | II | March, 2022  (estimated) | Multi-line | Recombinant human adenovirus type 5 injection | Recurrent cervical cancer | 55 | ORR | PFS, DCR, OS |
| Camrelizumab | NCT05151549 | II | December 1, 2021 | First-line | Cisplatin or carboplatin | Stage III-IVA PD-L1-positive cervical cancer with pelvic lymph nodes >2cm, positive para-aortic lymph nodes, or lymph node metastases >2 | 46 | PFS | DOR, PFS, OS, DCR |
| Cemiplimab | NCT04646005 | II | June 28, 2021 | Multi-line | ISA101b | Recurrent/metastatic HPV16 positive cervical cancer | 103 | ORR | TEAE, AE, SAE, SLAs, DOR, PFS, OS |
| Nivolumab | NCT02257528 | II | May 18, 2015 | Multi-line | / | Persistent, recurrent, or metastatic cervical cancer | 26 | ORR, SAE | PFS, OS |
| Nivolumab | NCT02465060 | II | August 12, 2015 | Multi-line | Targeted Therapy | Advanced refractory solid tumors, lymphomas, or multiple myeloma | 6452 | ORR | OS, PFS |
| Nivolumab | NCT02379520 | I | September, 2015 | NOS | Nivolumab, cytoxan, fludarabine | HPV-related cancer | 32 | DLT | ORR |
| Nivolumab | NCT03126110 | I/II | April 25, 2017 | NOS | Ipilimumab, INCAGN01876 | Advanced or metastatic malignancies | 145 | AE, ORR | ORR, DOR, DCR, PFS, OS, AE |
| Nivolumab | NCT03241173 | I/II | October 9, 2017 | First-line | Ipilimumab, INCAGN01949 | Advanced or metastatic malignancies | 52 | TEAE, ORR | ORR, DOR, DCR, DoDC, PFS, OS, TEAE |
| Nivolumab | NCT03220009 | II | November 3, 2017 | First-line | Ipilimumab, radiotherapy | High-risk localized, locoregionally advanced, or recurrent mucosal melanoma | 0 | RFS | DRFS, AE, OS, Rate of delayed surgery |
| Nivolumab | NCT03298893 | I/II | November 27, 2017 | NOS | Cisplatin, radiotherapy | Locally advanced cervical cancers | 21 | DLT | ORR, PFS, DFS, SAE, AE et al. |
| Nivolumab | NCT03508570 | I | September 21, 2018 | Multi-line | Ipilimumab | Recurrent or metastatic gynecologic cancer | 48 | RP2D, MTD | / |
| Nivolumab | NCT03527264 | II | November 8, 2018 | NOS | Cisplatin, radiotherapy | Advanced cervical cancer | 4 | DLT, PFS | / |
| Nivolumab | NCT04042116 | I/II | July 29, 2019 | NOS | Lucitanib | Advanced solid tumor | 227 | DLT, MTDs, ORR | AE, DOR, PFS, DCR, OS, PK, PD |
| Nivolumab | NCT04256213 | NA | July 2, 2020 | NOS | Ipilimumab | Cervical squamous cell carcinoma | 40 | CD8+/FOXP3+ ratio | AE, ORR, PFS, OS |
| Nivolumab | NCT04895709 | I/II | May 27, 2021 | Multi-line | BMS-986340 | Advanced solid tumors | 185 | AE, SAE, SLPs | PK, immunogenicity, ORR, DCR, DOR, PFS |
| Nivolumab | NCT04925284 | I | June 7, 2021 | Multi-line | XB002 | Advanced solid tumors (including persistent, recurrent, or metastatic cervical cancer) | 451 | MTD, ORR | AE, SAE, PK, DOR, PFS, OS |
| Nivolumab | NCT05180799 | I/II | January 30, 2022 | NOS | BA3071 | Advanced solid tumors | 36 | AE, SAE, ORR | PK, BOR, ORR, DCR, TTR, OS, DOR, PFS |
| Pembrolizumab | NCT02628067 | II | December 18, 2015 | Multi-line | / | Advanced, unresectable and/or metastatic solid tumors | 1595 | ORR | DOR, PFS, OS |
| Pembrolizumab | NCT02635360 | II | January 2016 | Multi-line | Brachytherapy, cisplatin | Advanced cervical cancer | 88 | IG, DLT | MRR, IDM, PFS, OS |
| Pembrolizumab | NCT02635672 | I | February 10, 2016 | Multi-line | VIP152 (BAY 1251152) | Advanced cancer | 110 | DLTs, PK, RP2D, AE | ORR |
| Pembrolizumab | NCT03108495 | II | June 22, 2017 | DDC | LN-145 | Recurrent, metastatic, or persistent cervical carcinoma | 138 | ORR, AE, Efficacy | DOR, DCR, PFS, ORR, AE, OS |
| Pembrolizumab | NCT03192059 | II | July 1, 2017 | Multi-line | Cyclophosphamide, aspirin, vitamin D, lansoprazole, curcumin, radiation | Advanced and/or refractory cervical cancer, endometrial carcinoma or uterine sarcoma | 43 | ORR | TEAEs, ORR, BOR, PFS, OS, QOL, |
| Pembrolizumab | NCT03277352 | I/II | November 21, 2017 | First-line | INCAGN01876, Epacadostat | Advanced or metastatic malignancies | 10 | TEAE, ORR, CRR | DCR, DOR, PFS, OS |
| Pembrolizumab | NCT03144466 | I | December 21, 2017 | First-line | Cisplatin, radiotherapy, brachytherapy | Locally advanced cervix cancers (stage IB-IVA) | 1 | MTD, PFS | Toxicities, ORR, HPV status, OS, PFS |
| Pembrolizumab | NCT03454451 | I | April 25, 2018 | Multi-line | Ciforadenant, CPI-006 | Select advanced cancers | 378 | DLT, TEAE, MDL | PK, ORR |
| Pembrolizumab | NCT03444376 | I/II | May 23, 2018 | First-line | GX-188E Vaccination | Advanced, non-resectable HPV 16/18-positive cervical cancer | 60 | DLT, ORR | ORR, BOR, TTR, DOR, PFS, OS |
| Pembrolizumab | NCT03236935 | I | August 3, 2018 | Multi-line | L-NMMA | Unresectable or metastatic TMB-H solid tumors | 12 | MTD | DLT, RP2D, Antitumor activity, PK |
| Pembrolizumab | NCT03367871 | II | September 6, 2018 | First-line | Bevacizumab, Paclitaxel, Cisplatin/Carboplatin | Recurrent, persistent, or metastatic cervical cancer | 40 | ORR | PFS, OS, AE, SAE |
| Pembrolizumab | NCT03674567 | I/II | September 25, 2018 | NOS | FLX475 | Advanced cancer (including cervical cancer) | 375 | AE, DLTs, MTD | ORR |
| Pembrolizumab | NCT03635567 | III | October 25, 2018 | First-line | Bevacizumab, Paclitaxel, Cisplatin/Carboplatin | Persistent, recurrent, or metastatic cervical cancer | 600 | PFS, OS | ORR, DOR, PFS, AE, SAE, irAE, QOL |
| Pembrolizumab | NCT03755739 | II/III | November 1, 2018 | NOS | Ipilimumab | Advanced solid tumor | 200 | OS, CRR | PFS, DOR, DCR, COD |
| Pembrolizumab | NCT03799003 | I | January 14, 2019 | Multi-line | ASP1951 | Locally advanced, unresectable or metastatic solid tumors | 435 | DLT, AE, irAE, IRRs, SAE, ECG, H&P abnormalities, ECOG, PK | ORR, DOR, DOR, DCR |
| Pembrolizumab | NCT03476681 | I/II | January 18, 2019 | NOS | NEO-201 | PD-L1-positive, MSI-H, or TMB-H cervical cancer |  | AE, ORR, PFS | AE, PK |
| Pembrolizumab | NCT03786081 | I/II | February 27, 2019 | DDC | Tisotumab Vedotin, bevacizumab, carboplatin | Recurrent or stage IVB cervical cancer | 175 | DLT, ORR | AE, ORR, DOR, TTR, PFS, OS |
| Pembrolizumab | NCT03917381 | I/II | May 14, 2019 | NOS | GEN1046 | Refractory, advanced and/or metastatic solid tumors (including cervical cancer) | 572 | DLT, AE, SLPs, ORR | ORR, PK, DCR, DOR, AE, SLPs, OS |
| Pembrolizumab | NCT03849469 | I | May 29, 2019 | Multi-line | XmAb®22841 | Histologically or cytologically confirmed advanced or metastatic solid tumors | 242 | TEAE | / |
| Pembrolizumab | NCT04234113 | I | June 13, 2019 | Multi-line | SO-C101 | Advanced/metastatic solid tumors (including cervical cancer) | 200 | DLT, AE, SAE, PS | PK, ORR, BOR, DOR, CBR, PFS, immunogenicity |
| Pembrolizumab | NCT04099277 | I | October 28, 2019 | NOS | LY3435151 | Advanced solid tumors (including cervical cancer) | 2 | DLTs, AE | PK, ORR, DCR, DOR, TTR, PFS, |
| Pembrolizumab | NCT04187872 | I | January 10, 2020 | NOS | Laser interstitial thermot-therapy | Primary cancer with recurrent brain metastasis after prior stereotactic radiosurgery | 16 | Immune profile of PBMCs | TEAEs |
| Pembrolizumab | NCT04238988 | II | March 1, 2020 | NOS | Carboplatin, paclitaxel | Stage IB2-IIB cervical cancer | 45 | PFS | OS, RR, AE, QOL, IRGS |
| Pembrolizumab | NCT04230954 | II | April 16, 2020 | First-line | Cabozantinib | Recurrent, persistent and/or metastatic cervical cancer | 39 | PFS | ORR, OS, AE, SAE, QOL |
| Pembrolizumab | NCT04221945 | III | May 12, 2020 | First-line | Cisplatin, EBRT, Brachytherapy | Locally advanced cervical cancer | 980 | PFS, OS | PFS, OS, CRR, ORR, QOL, AE |
| Pembrolizumab | NCT04301011 | I/II | June 2, 2020 | NOS | TBio-6517 | Advanced solid tumors (including cervical cancer) | 138 | AE, MTD, ORR | AE, OS, DOR, ORR, DCR, TTP, PFS |
| Pembrolizumab | NCT04485013 | I | July 14, 2020 | NOS | TTX-080, cetuximab | Advanced refractory/resistant solid malignancies | 200 | ORR | DOR, PFS, OS, AE, PD, PK |
| Pembrolizumab | NCT04483544 | II | August 10, 2020 | First-line, second-line | Olaparib | Advanced or recurrent cervical carcinoma | 48 | ORR | PFS, TEAE, DOR |
| Pembrolizumab | NCT04432857 | I | August 20, 2020 | DDC | AN0025 | Locally advanced and nonresectable, or metastatic disease | 84 | DLT | ORR, PFS, DOR, OS, efficacy |
| Pembrolizumab | NCT04140526 | I/II | September 16, 2020 | NOS | ONC-392 | Advanced or metastatic solid tumors | 468 | DLT, MTD, RP2D, TRAE | PK, ORR, PFS, OS |
| Pembrolizumab | NCT04357873 | II | October 28, 2020 | Multi-line | Vorinostat | Recurrent and/or metastatic squamous cell carcinoma | 112 | ORR | ORR, DOR, PFS, OS |
| Pembrolizumab | NCT04652076 | I/II | December 14, 2020 | Second-line, third-line | NP137, paclitaxel, carboplatin | Locally advanced/metastatic endometrial carcinoma or cervix carcinoma | 240 | DLT, ORR | CBR, DOR, PFS, OS, DOR, PK, PD |
| Pembrolizumab | NCT04641728 | II | January 1, 2021 | NOS | Olaparib | Recurrent or metastatic cervical cancer | 28 | ORR | ORR, DOR, DRR, PFS, TEAE |
| Pembrolizumab | NCT04913337 | I/II | June 9, 2021 | Multi-line | NGM707 | Advanced or metastatic solid tumors | 179 | DLT, AE, SLAs, ORR, DOR, PFS, OS | PK |
| Pembrolizumab | NCT05215574 | I | March 31, 2022 | Multi-line | NGM831 | Advanced or metastatic solid tumors | 79 | DLT, AE, SLAs | PK, ORR |
| Pembrolizumab | NCT05311618 | I | May 11, 2022 | Multi-line | NGM438 | Advanced or metastatic solid tumors | 71 | DLT, AE, SLAs, PD | PK, ORR |
| Pembrolizumab | NCT04712851 | II | June 30, 2021 | NOS | / | Cervical intraepithelial neoplasia, cervical squamous cell carcinoma in situ, cervical squamous intraepithelial neoplasia II | 25 | PRR | PPR, AE |
| Pembrolizumab | NCT05098132 | I | January 25, 2022 | Multi-line | STK-012 | Selected solid tumor (including cervical cancer) | 135 | DLT, AE | ORR, PFS, OS, PD, PK, immunogenicity |
| Pembrolizumab | NCT05269381 | I | March 31, 2022 | Multi-line | Cyclophosphamide, neoantigen peptide vaccine, pembrolizumab, sargramostim | Advanced solid tumors (including cervical cancer) | 36 | AE | Immunogenicity, ORR |
| Pembrolizumab | NCT05082259 | I | March 2, 2022 | Multi-line | ASTX660 | Advanced cervical cancer, refractory to conventional treatment | 48 | MTD, RP2D, AE, ORR, BOR, PFS, DOR | ORR, BOR, PFS, DOR, PK |
| Pembrolizumab | NCT04865887 | II | June 2022 (estimated) | Multi-line | Lenvatinib | Locally advanced or metastatic cervical cancer | 35 | ORR | DOR, PFS, OS, AE, SAE |
| Pembrolizumab | NCT05438420 | I/II | October 30, 2022  (estimated) | Multi-line | Q702 | Selected advanced solid tumors (including cervical cancer) | 120 | TEAEs, ORR | PK |
| Pembrolizumab | NCT05259540 | II | January 1, 2023  (estimated) | NOS | Compound kushen injection | Metastatic, or recurrent, persistent cervical adenocarcinoma | 42 | ORR | PFS, DCR, DOR |
| MK-7684A (pembrolizumab/ vibostolimab) | NCT05007106 | II | September 16, 2021 | NOS | Chemotherapy (cisplatin, paclitaxel et al.) | Selected solid tumors (including cervical cancer) | 610 | ORR, PFS | OS, PFS, DOR, ORR, QOL, AE, SAE |
| Sindilimab | NCT04799639 | II | March, 2021  (estimated) | First-line | Paclitaxel, cisplantin | Clinical FIGO stage IB3 or IIA2 cervical cancer | 47 | pCR | ORR |
| Sintilimab | NCT04096911 | II | July 31, 2019 | Second-line | Quadrivalent HPV vaccine | Persistent, recurrent or metastatic cervical cancer | 20 | ORR | PFS, OS, DOR |
| Sintilimab | NCT04918628 | II | May 1, 2021 | NOS | / | Stage IIIC2-IVB cervical cancer | 50 | TRAE | PFS, OS, occurrence of IC/IR/ABP |
| Sintilimab | NCT05383482 | I/II | June 30, 2022 | Multi-line | Afuresertib, nab paclitaxel, docetaxel | Selected solid tuomrs (including cervical cancer) that resistance to prior anti-PD-1/PD-L1 | 167 | I:AE,DLT,RP2D  II: ORR | I: ORR, DCR, DOR, PFS, PK  II: OS |
| Spartalizumab | NCT04802876 | II | April 12, 2021 | NOS | / | PD1-high-expressing tumors | 141 | ORR | CBR, PFS, DOR, TTR, OS, TEAE |
| Serplulimab | NCT05444374 | II | October 1, 2022  (estimated) | First-line | Bevacizumab, cisplatin, paclitaxel | Untreated recurrent or metastatic cervical cancer | 48 | ORR | PFS, DOR, DCR, OS, AE |
| Tislelizumab | NCT04693234 | II | March 3, 2021 | Multi-line | Ociperlimab | Recurrent or metastatic cervical cancer | 167 | ORR | ORR, DOR, PFS, TTR, DCR, CBR, OS, QOL, AE, SAE |
| Tislelizumab | NCT05013268 | I | September 2021 | Neoadjuvant | Paclitaxel, cisplatin or carboplatin | IB2-IIB cervical cancer | 15 | MPR | pCR, ORR, RFS, DFS, AE, OS |
| Toripalimab | NCT04368273 | I/II | May 8, 2020 | First-line | Radical radiotherapy concurrent chemotherapy | Locally advanced cervical cancer | 30 | AE | ORR, PFS |
| Toripalimab | NCT04395612 | II | May 8, 2020 | Multi-line | Niraparib, brivanib | Metastatic, recurrent and persistent cervical cancer | 38 | ORR | PFS, DCR |
| Toripalimab | NCT04651127 | I/II | November 9, 2020 | Multi-line | Chidamide | Persistent, recurrent or metastatic cervical cancer | 40 | DLT, ORR | PFS, DOR, DCR, OS, AE |
| Toripalimab | NCT05084677 | II | January 1, 2021 | First-line | Concurrent platinum-based chemoradiotherapy | Locally advanced cervical cancer | 96 | ORR | PFS, OS |
| Toripalimab | NCT04731038 | I | July 1, 2021  (estimated) | NOS | Anlotinib, Paclitaxel, Cisplatin/ Carboplatin | Persistent, recurrent or metastatic cervical cancer | 20 | AE | PFS, OS, ORR, DCR, DOR |
| Toripalimab | NCT04973904 | II | August 1, 2021 | First-line | Paclitaxel, Cisplatin, Bevacizumab | Advanced cervical cancer | 35 | ORR | DCR, PFS, OS |
| Toripalimab | NCT05342506 | II | April 18, 2022 | Multi-line | ScTIL | Recurrent or refractory cervical cancer | 30 | ORR | DCR, DOR, PFS, OS |
| **PD-L1 Antibody** |  |  |  |  |  |  |  |  |  |
| AK112 | NCT04870177 | II | April 9, 2021 | NOS | / | Advanced gynecological tumors | 270 | ORR | DCR, DOR, TTR, PFS, OS, PK et al. |
| M7824 | NCT03427411 | II | February 27, 2018 | NOS | / | Locally advanced or metastatic HPV-associated malignancies | 57 | ORR | DOR, OS, PFS, DCR, AE, RR |
| M7824 | NCT04551950 | I | October 19, 2020 | First-line | Bevacizumab, Paclitaxel, Cisplatin/Carboplatin, Radiotherapy | Locally advanced or advanced cervical cancer | 25 | DLT, AE | PK, PD, IG, DLT, AE |
| M7824 | NCT04432597 | I/II | August 11, 2020 | Multi-line | PRGN-2009 | Hpv-positive cancer | 76 | Safety and RP2D, IG | ORR, PFS, OS, DOR, AE, IG |
| M7824 | NCT04246489 | II | March 30, 2020 | Multi-line | / | Advanced, unresectable cervical cancer | 146 | ORR | DOR, TEAE, PFS, OS, PK |
| MT-6402 | NCT04795713 | I | May 27, 2021 | NOS | / | Advanced solid cancer that expresses PD-L1 | 138 | MTD, AE, RP2D, ORR | PF, ORR, DOR, PFS, OS, immunogenicity |
| TQB2450 | NCT04623333 | II | November 30, 2020 | Multi-line | / | PD-L1 positive recurrent or metastatic cervical cancer | 80 | ORR | ORR, DCR, DOR, PFS, OS |
| TQB2858 | NCT05068921 | I | December 9, 2021 | NOS | / | Advanced cervical cancer | 40 | ORR | PFS, DCR, DOR, OS, AE, SAE, TEAEs |
| ZKAB001 | NCT03676959 | I | August 16, 2018 | Second-line | / | Recurrent or metastatic cervical cancer | 101 | RP2D, ORR, TRAE | IG, PFS, OS, DOR, BOR |
| Atezolizumab | NCT02914470 | I | January, 2017 | First-line, second-line | Carbplatin, cyclophophamide | Advanced breast cancer and gynaecologic cancer | 12 | AE | ORR |
| Atezolizumab | NCT02921269 | II | March 10, 2017 | Second-line, third-line | Bevacizumab | Recurrent, persistent or metastatic cervical cancer | 11 | ORR | PFS, OS, AE, PD-L1 expression, IG |
| Atezolizumab | NCT03073525 | II | May 25, 2017 | NOS | Vigil | Advanced gynecological cancers | 25 | TEAE | Immune RR, RTA, TTP |
| Atezolizumab | NCT03340376 | II | August 30, 2017 | Second-line | Doxorubicin | Recurrent cervical cancer | 48 | PFS | OS |
| Atezolizumab | NCT03386721 | II | February 19, 2018 | Multi-line | Simlukafusp alfa, gemcitabine, vinorelbine | Advanced and/or metastatic solid tumors | 256 | ORR | DCR, DOR, PFS, OS, AE, IG |
| Atezolizumab | NCT03612791 | II | August 13, 2018 | First-line | Cisplatin, radiotherapy | Locally advanced cervical cancer | 189 | PFS | / |
| Atezolizumab | NCT03556839 | III | September 25, 2018 | First-line | Bevacizumab, Paclitaxel, Cisplatin/Carboplatin | Metastatic, persistent or recurrent cervical cancer | 404 | OS | PFS, ORR, DOR, TEAE, QOL, PK, IG |
| Atezolizumab | NCT03738228 | I | October 26, 2018 | First-line | Radiotherapy, brachytherapy, cisplatin | Node-positive locally advanced cervical cancer | 40 | IG | DLT, AE, IG, PD-L1 expression |
| Atezolizumab | NCT03829501 | I/II | January 28, 2019 | NOS | KY1044 | Selected advanced malignancies | 412 | AE, SAE, ORR, DLT | BOR, PFS, OS, DOR, ORR, AE, SAE |
| Atezolizumab | NCT03614949 | II | January 29, 2019 | NOS | SBRT | Recurrent, persistent, or metastatic cervical cancer | 26 | ORR | PFS, OS |
| Atezolizumab | NCT03946358 | II | February 18, 2020 | Multi-line | UCPVax | HPV-positive locally advanced or metastatic cervical SCC | 47 | ORR | OS, PFS, QOL |
| Atezolizumab | NCT04300647 | II | June 30, 2020 | Multi-line | Tiragolumab | Metastatic and/or recurrent pd-l1-positive cervical cancer | 160 | ORR | AE, DOR, DCR, BCR, PFS, OS, PK |
| Atezolizumab | NCT04405349 | II | July 1, 2020 | NOS | HPV vaccine (VB10.16) | Advanced or recurrent non-resectable HPV16-positive cervical cancer | 50 | AE, ORR | DOR, PFS, OS, IG |
| Avelumab | NCT03260023 | I/II | September 11, 2017 | Multi-line | TG4001 | HPV-16 positive advanced, recurrent or metastatic malignancies | 150 | DLT, ORR, PFS | ORR, PFS, OS, DOR, DCR, AE |
| Durvalumab | NCT01975831 | I | December 19, 2013 | NOS | Tremelimumab | Advanced solid tumors | 104 | AE | BOR, PFS, OS |
| Durvalumab | NCT02291055 | I/II | April, 2015 | First-line | ADXS11-001 | Locally advanced or metastatic cervical cancer | 66 | AE, PFS | / |
| Durvalumab | NCT02725489 | II | June 3, 2016 | NOS | Vigil | Locally advanced or metastatic women's cancers | 13 | TEAE | ORR, Disease status, IFNγ-ELISPOT conversion rate |
| Durvalumab | NCT03277482 | I | February 6, 2018 | Multi-line | Tremelimumab | Recurrent or metastatic gynecologic cancer | 32 | MTD | ORR, LRR, LCR, DOR, PFS, OS |
| Durvalumab | NCT03518606 | I/II | June 20, 2018 | NOS | Tremelimumab, metronomic vinorelbine | Locally advanced or metastatic solid tumours | 150 | MTD, RP2D, CBR | / |
| Durvalumab | NCT03452332 | I | July 18, 2018 | Multi-line | Tremelimumab, SBRT | Recurrent or metastatic cervical, vaginal, or vulvar cancers | 20 | AE | RTT, PFS, OS, TTNT |
| Durvalumab | NCT03439085 | II | November 14, 2018 | First-line | MEDI0457 | Recurrent or metastatic HPV associated cancers | 77 | ORR | ORR, DCR, PFS, OS |
| Durvalumab | NCT03830866 | III | February 15, 2019 | First-line | Cisplatin, Carboplatin, EBRT, brachytherapy | Locally advanced cervical cancer | 770 | PFS | OS, CRR, ORR, DOR, QOL |
| Durvalumab | NCT03983954 | I | October 10, 2019 | Multi-line | Obinutuzumab, naptumomab estafenatox (ABR-217620) | Selected advanced or metastatic solid tumors | 45 | AE, DLT, RP2D | ORR, DOR, PFS, OS, RP2D, IG |
| Durvalumab | NCT04504669 | I | August 18, 2020 | First-line | AZD8701 | Select advanced solid tumors | 123 | MTD, PR2D, AE, SAE, ORR | PFS, DOR, DCR, TTR, CTS, OS, PK, PD, FOXP3 mRNA expression |
| Durvalumab | NCT04800978 | II | June 14, 2021  (estimated) | Second-line | BVAC-C | HPV16/18 positive cervical cancer | 37 | DLT, PFS | BOR, DCR, PFS, OS, AE |
| **Combined PD-1/PD-L1 Antibody** | |  |  |  |  |  |  |  |  |
| Nivolumab, Pembrolizumab, Atezolizumab | NCT03841110 | I | February 15, 2019 | NOS | FT500, Cyclophosphamide, Fludarabine, IL-2 | Advanced solid tumors | 76 | DLT | ORR, DOR |
| Nivolumab, Pembrolizumab, Atezolizumab, Avelumab | NCT03228667 | II | December 11, 2018 | Multi-line | N-803 | Advanced, recurrent or metastatic carcinoma | 145 | ORR | DSF, OS, TTR, DOR, AE, QOL, PFS |
| Nivolumab, Pembrolizumab, Atezolizumab, Durvalumab | NCT03544723 | II | October 1, 2018 | NOS | Adenoviral p53 | Recurrent or metastatic solid tumors and lymphoma | 40 | ORR, AE | DOR, PFS |
| Pembrolizumab, durvalumab | NCT05187338 | I/II | November 1, 2021 | NOS | Ipilimumab | Advanced solid tumors | 100 | AE, PFS, DCR, DOR | OS |

**Abbreviations:** ABP, abnormal blood pressure; AE, adverse events; BOR, best overall response; CBR, clinical benefit response; CIN, cervical intraepithelial neoplasia; CPI-006, a humanized monoclonal antibody targeting the CD73 cell-surface ectonucleotidase; CRR, complete response rate; CTS, change in tumour size; DCR, disease control rate; DDC, determined by different cohorts; DFS, disease free survival; DLT, dose limiting toxicities; DoDC, duration of disease control; DOR, duration of response; DRFS, distant recurrence-free survival; EBRT, external beam radiotherapy; FIGO, International Federation of Gynecology and Obstetrics; FT500, an allogeneic, iPSC-derived natural killer cell cancer immunotherapy; H&P, history and physical examination; HPV, human papillomavirus; IC, immunological colitis; IDM, incidence of distant metastases; IG, immunogenicity; IR, immunological rash; irAE, immune-related adverse event; IRGS, immune-related gene signatures; IRRs, infusion-related reactions; LCR, local control rate; L-NMMA, NG-monomethyl-L-arginine; LRR, local response rate; MDL, maximum dose level; MPR, major pathological response; MRR, metabolic response rate on PET/CT imaging; MSI-H, microsatellite instability-high; MTD, maximum tolerated dose; NOS, not otherwise specified; ORR, overall response rate; OS, overall survival; PBMC, peripheral blood mononuclear cells; pCR, pathological complete response; PD, pharmacodynamics; PFS, progression-free survival; PK, pharmacokinetics; PPR, pathologic partial response; PROs, patient reported outcomes; PRR, pathological response rate; PR2D, the phase II recommended dose; PS, performance status; QOL, quality of life; RD, remission duration; RFS, recurrence-free survival; RTA, radiological tumor assessment; RTT, response to treatment; SAE, severe adverse events; SCC, squamous cell carcinoma; SLAs, ≥grade 3 laboratory abnormalities or significant laboratory abnormalities; SLPs, safety laboratory parameters (hematology, biochemistry, coagulation, endocrines); TCR, T-cell receptor; TCRBCE, T cell receptor beta clonal expansion; TEAEs, treatment-emergent adverse events; TMB-H, tumor mutational burden-high; TTNT, time to next treatment; TTP, time to progression; TTR, time to response; Vigil, engineered autologous tumor cell immunotherapy.
